# MicroRNAs as the promising markers of comorbidities in childhood obesity—A systematic review

**DOI:** 10.1111/ijpo.12880

**Published:** 2021-12-16

**Authors:** Michał Hutny, Jagoda Hofman, Agnieszka Zachurzok, Paweł Matusik

**Affiliations:** ^1^ Scientific Society of Medical Students, Faculty of Medical Sciences in Katowice Medical University of Silesia Katowice Poland; ^2^ Department of Pediatrics, Faculty of Medical Sciences in Zabrze Medical University of Silesia Katowice Poland; ^3^ Department of Pediatrics, Pediatric Obesity and Metabolic Bone Diseases, Chair of Pediatrics and Pediatric Endocrinology, Faculty of Medical Sciences in Katowice Medical University of Silesia Katowice Poland

**Keywords:** children, epigenetics, microRNA, obesity

## Abstract

**Introduction:**

Rising child obesity rate creates a need for tools quantifying changes in children suffering from obesity, for purposes of detection or prevention of comorbidities. A candidate for such a role seems to be microRNAs, which in vivo serve as the suppressing factors in gene expression.

**Objectives:**

This study aimed at reviewing recent discoveries in this field and concluding directions of research or application of studied molecules.

**Methods:**

Repeated browsing of databases and screening of results, led to final approval of 16 articles. Filtered studies examined differences in microRNA expression between subjects with obesity and children suffering from its comorbidities.

**Results:**

Studies concerning endothelial dysfunction identified molecules miR‐320a and miR‐630 as a possible diagnosis and treatment option. Search for the alternative markers in diagnosis of non‐alcoholic fatty liver disease suggested value of molecules: miR‐199a‐5p and miR‐122. miR‐486, miR‐146b, and miR‐15b may serve in grading the development of type 2 diabetes in children, although further research raised doubts. Panel of molecules was indicated as useful in early detection of metabolic syndrome and insulin resistance associated alterations. No valid link between studied microRNAs and atherosclerosis was found.

**Conclusions:**

MicroRNAs seem to be promising prognostic markers for diagnosis of endothelial dysfunction, non‐alcoholic fatty liver disease, type 2 diabetes, metabolic syndrome and insulin resistance in children.

**Abbreviations**
ABCA1: ATP‐binding cassette transporters A1
ABCG1: ATP‐binding cassette transporters G1
AMP: adenosine monophosphate
AS: atherosclerosis
AUC: area under the receiver–operator curve
BMI: body‐mass index
BMI‐z: body‐mass index Z‐score
C2C12: myoblasts culture
CG: control group
CGS: WHO Child Growth Standards
CK18: cytokeratin 18
db: mice with diabetes
ED: endothelial dysfunction
EG: experimental group
ELISA: enzyme‐linked immunosorbent assay
ET: endothelin
EV: extracellular vesicle
FPG: fasting plasma glucose
GC: German cohort
HbA1: glycated haemoglobin
HbA1c%: measurement of percentage of HbA1c
HOMA‐IR: homeostatic model assessment insulin resistance
IC: Italian cohort
ICAM‐1: intercellular adhesion molecule 1
IR: insulin resistance
MALAT1: metastasis‐associated lung adenocarcinoma transcript 1
MetS: metabolic syndrome
MIN6: murine beta‐cells culture
miR: microRNA
miRNA: microRNA
mRNA: messenger ribonucleic acid
NAFLD: non‐alcoholic fatty liver disease
NASH: non‐alcoholic steatohepatitis
NEF: normal endothelial function
NO: nitric oxide
NW: patients without obesity
OB: children with obesity
ob: mice with obesity
OW: children who were overweight
PAT: peripheral arterial tonometry
PICOS: participants, interventions, comparisons, outcomes, study design
RHI: reactive hyperaemic index
RNA: ribonucleic acid
ROC: receiver–operator curve
SC: Slovenian cohort
SD: standard deviation
T2DM: diabetes mellitus
Tmax: time of peak reperfusion
USG: ultrasonography
VAT: visceral adipose tissue
VCAM‐1: vascular cell adhesion molecule 1

## 1 INTRODUCTION

Obesity in children is one of the most demanding challenges emerging in the modern world of paediatric medicine. It is described in relation to patient's body‐mass index (BMI). In children a threshold of obesity is BMI above 95th percentile in a group of the same age and sex. Despite various prevention programs, analyses, and research, its occurrence is still high,^1^ which exposes youth to complications of obesity, such as endothelial dysfunction (ED),^2^ non‐alcoholic fatty liver disease (NAFLD),^3^ type 2 diabetes mellitus (T2DM),^4^ atherosclerosis (AS),^5^ and obstructive sleep apnea.^6^ The possibility to quantify the progression of these diseases is necessary in order to overcome their development. As the standard markers and tests in many cases are insufficient measures for this challenge, the novel markers such as microRNA (miRNAs; miRs) may prove to be a sound solution. MiRNAs are small, non‐coding molecules that play a suppressive role in expression of genes by binding to 3′UTR end of their target messenger ribonucleic acid (mRNA). They can be found in body tissues or fluids, like saliva, plasma, serum, or whole blood; encapsulated into extracellular vesicles (EVs) or as circulating miRNA.^7^, ^8^ Changes in a profile of the circulating and tissue miRNAs directly influence the physiology of tissues and cells involved in glucose and lipid metabolism – pancreatic β‐cells, hepatocytes, skeletal muscle tissue, and adipose tissue – by modulating mRNA translation.^9^, ^10^ Taking into consideration the easy accessibility and measurability of materials rich in miRNAs, such as blood, plasma, and saliva,^11^, ^12^, ^13^ examining the associations between diseases and miRNA levels might lead to creation of diagnostic and prognostic markers of high sensitivity and specificity.

Research on miRNA is nowadays one of the more popular scopes of interest in numerous branches of medicine. Recently the differences in miRNA profiles between the children with and without obesity have been investigated.^14^, ^15^ Results of studies on this topic were analysed and summarized into the systematic review in 2019 by Oses et al. It also presented two miRNAs significantly overexpressed in children with NAFLD and T2DM.^16^ Our study concentrated solely on reviewing studies that examined miRNAs as biomarkers of paediatric obesity comorbidities. The scope of interest was expanded to comorbidities other than NAFLD and T2DM. The set of included studies was updated. In accordance with ‘participants, interventions, comparisons, outcomes, and study design (PICOS)’ scheme, the following question was created for the purpose of review: ‘What are the possible applications of miRNAs in diagnosis, prediction and treatment of obesity comorbidities in paediatric population?’ Results of 16 studies on miRNAs as markers of paediatric obesity comorbidities were enrolled (only 3 of which overlap with the previous review), including not only T2DM and NAFLD, but also ED, metabolic syndrome, insulin resistance, and AS. More than a half of the studies included in this review were published after the last date of literature search conducted in the previous review (23 November 2018). The time range in which the included studies of current review were published was 2016–2021.

## 2 MATERIALS AND METHODS

Entry terms were formulated according to the following scheme:

- Subject group: ‘child obesity’ OR ‘childhood obesity’ OR ‘obese children’ OR ‘paediatric obesity’ OR ‘overweight children’ OR ‘adolescent obesity’

AND

- Comorbidity: ‘complications’ OR ‘comorbidities’ OR ‘NAFLD’ OR ‘non‐alcoholic fatty liver disease’ OR ‘liver’ OR ‘fatty liver’ OR ‘CVD’ OR ‘cardiovascular diseases’ OR ‘endothelial dysfunction’ OR ‘type 2 diabetes’ OR ‘hypertension’ OR ‘metabolic syndrome’ OR ‘puberty’ OR ‘asthma’ OR ‘obstructive sleep apnea’ OR ‘‐’

AND

- Marker: ‘miRNA’ OR ‘miR’ OR ‘microRNA’ OR ‘circulatory microRNA’

Adequate filters regarding publication dates (up to 5 years prior to search) and text availability were applied. The repeated results of search were ruled out.

Retrieved data underwent further screening on a basis of titles, abstracts, and keywords (performed by MH and JH) according to inclusion and exclusion criteria, which led to exclusion of reviews, systematic reviews, author manuscripts, personal communication, letters to editor, conference material, and case reports. Articles concerning the miRNA as the markers of obesity itself in children, as well as the studies of miRNA not obtained from blood or plasma were ruled out – in order to be plausible for the role of markers, miRNAs must be easily accessible (blood or plasma) and correlate with comorbidities of obesity (rather than the obesity itself). Inadequate matching of experimental and control groups in the matter of age or exceeding upper age threshold were also the discriminating factor.

At the stage of data synthesis the retrieved studies were assessed by two independent researchers (AZ and PM) in terms of methodology quality, as well as relevance of the results. The principal measures of differentiating potential were area under the receiver–operator curve (ROC) (AUC) score, fold change, and *p*‐values (statistical significance threshold: *p* < 0.05).

## 3 RESULTS

Multiple browsing of databases, such as PubMed, askMEDLINE, Wiley Online Library, and Elsevier, conducted in the span of January 2020 – September 2021 using search strategy presented in Materials and Methods section, returned a total of 1754 results. That number was reduced to 245 individual articles by excluding 1509 repetitions. The remaining have been found eligible for further title and abstract valuation, of which 34 studies were accepted as fitting for whole text reading according to the inclusion and exclusion criteria, which were as follows:

Inclusion criteria:

- English literature published in the last 5 years
- Studies in human subjects
- Paediatric population (<19 yo)
- Studies examining miRNA profiles of patients with obesity

Exclusion criteria:

- Publication type: reviews, systematic reviews, author manuscripts, personal communication, letters to editor, conference material, and case report
- Studies based on miRNA from body fluids other than blood
- Studies not linking miRNA of patients suffering from obesity to any comorbidity
- Subjects exceeding at the study time the age criterion of paediatric group (≥19 yo)
- Control group inadequate in terms of age to the study group
- Subjects diagnosed with comorbidity other than the examined one

In total 16 studies fully met the inclusion and exclusion criteria.

**FIGURE 1:**
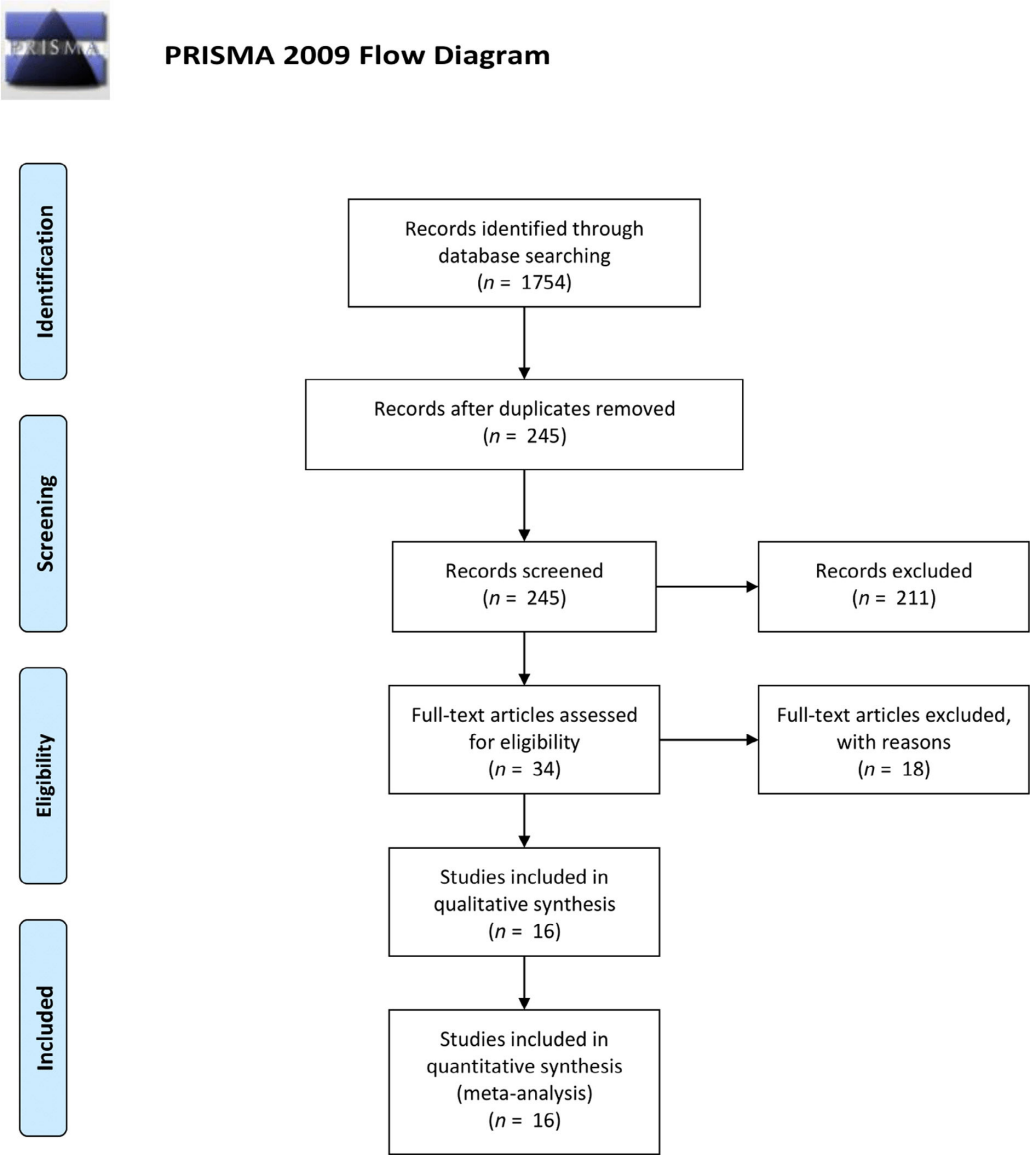
Flow diagram of research process. *Source*: Reference 17

The process of data collection for this review is presented in Figure 1.

Main characteristics of selected articles can be found in Table 1.

**TABLE 1:** Summary of article characteristics

Number	Authors	Year	Complication of obesity	Number of subjects	Molecules investigated	Sample source	Evaluation technique
1.	Donghui et al.^18^	2019	ED	67	miR‐126	Serum	RT‐qPCR
2.	Zhao et al.^19^	2021	ED	60	miR‐320a	Serum	RT‐qPCR
3.	Khalyfa et al.^20^	2016	ED	70	miR‐125a‐5p, ‐342‐3p, ‐365b‐3p	Plasma	RT‐qPCR, Microarrays
4.	Khalyfa et al.^21^	2016	ED	69	miR‐16‐5p, ‐451a, ‐5100, ‐630, ‐4665‐3p	Serum EVs	RT‐qPCR, Microarrays
5.	Thompson et al.^22^	2017	NAFLD	30	miR‐15b‐5p, ‐199a‐5p, ‐222‐3p, ‐223‐5p, ‐34a‐5p, ‐122‐5p, ‐23a‐5p, ‐27b‐3p, ‐21‐5p, ‐34a‐5p, ‐451‐5p, ‐192‐5p, ‐16‐5p, ‐29a‐3p, ‐150‐5p, ‐214‐3p, ‐375, ‐155‐5p, ‐191‐5p, ‐103a‐5p	Plasma	RT‐qPCR
6.	Brandt et al.^23^	2017	NAFLD	147	miR‐122	Serum/plasma	RT‐qPCR
7.	Cui et al.^24^	2018	T2DM	718	miR‐486, ‐146b, ‐15b	Serum	RT‐qPCR
8.	Mohany et al.^25^	2020	T2DM	120	miR‐486, ‐146b, ‐15b	Plasma	RT‐qPCR
9.	Mohany et al.^26^	2021	T2DM	298	miR‐29a, ‐122	Serum	RT‐qPCR
10.	Al‐Rawaf^27^	2020	MetS	250	miR–142‐3p, ‐140‐5p, ‐222, ‐143, ‐130, ‐532‐5p, ‐423‐5p, ‐520c‐3p, ‐146a, ‐15a	Plasma	RT‐qPCR
11.	Zhang et al.^28^	2021	MetS	147	miR‐24‐3p	Serum	RT‐qPCR
12.	Lischka et al.^29^	2021	MetS	109	miR‐15a, ‐19, ‐23a, ‐27b, ‐33a, ‐33b, ‐34a, ‐93, ‐98, ‐122, ‐144‐3p, ‐144‐5p, ‐192, ‐193b, ‐197, ‐1290	Serum	RT‐qPCR
13.	Lin et al.^30^	2020	IR	33	miR‐30d‐5p, ‐122‐5p, ‐221‐3p, ‐215‐5p	Serum	RT‐qPCR
14.	Yu et al.^31^	2018	IR	90	miR‐27a	Serum	RT‐qPCR
15.	Iacomino et al.^32^	2021	IR	58	miR‐191‐3p, ‐375	Serum	RT‐qPCR
16.	Barberio et al.^33^	2019	AS	93	miR‐3129‐5p, ‐20b, ‐9‐5p, ‐320d, ‐301a‐5p, ‐155‐5p	Circulating adipocyte‐derived EVs	Microarrays

Abbreviations: AS, atherosclerosis; ED, endothelial dysfunction; EVs, extracellular vesicles; IR, insulin resistance; MetS, metabolic syndrome; miR, miRNA; NAFLD, non‐alcoholic fatty liver disease; RT‐qPCR, reverse‐transcription quantitative polymerase chain reaction; T2DM, type 2 diabetes mellitus.

Enrolled studies focused on associations between changes in miRNA profiles of patients with obesity and presence of 6 obesity comorbidities. Their methodology is discussed in the following sections separately for each complication.

### 3.1 Endothelial dysfunction

The highest number of articles was linked to ED – a total of four studies (articles 1.; 2.; 3.; 4.), which examined among others the miRNAs: miR‐126, miR‐320a, miR‐125a‐5p, miR‐342‐3p, miR‐365b‐3p, and miR‐630. ED is a pathological state characterized by impaired maintenance of vascular tone and oxidative stress, resulting from dysregulation of synthesis of endothelial mediators – nitric oxide (NO), endothelin (ET), and others.^34^, ^35^ Patients were classified into groups of either normal endothelial function (NEF) or ED, based on their reactive hyperemic index (RHI) score. RHI was measured with peripheral arterial tonometry (PAT) (study 1) or with time of peak reperfusion (Tmax) after occlusion of arteries (studies 3 and 4). Condition of epithelium of patients in study 2 was inspected using levels of intercellular adhesion molecule 1 (ICAM‐1), vascular cell adhesion molecule 1 (VCAM‐1), and E‐selectin.^36^, ^37^, ^38^ Patients' characteristics are presented in Table 2.

**TABLE 2:** Articles concerning the differences of miRNA levels in endothelial dysfunction and physiology

Article	Group	Number of patients	Age (years)	Sex (% of male)	BMI‐z
					*BMI
1.	CG^a^	*n* = 10	12–18	100	Pre *23.05 ± 0.46
					Post *23.01 ± 0.50
	EG^a^	*n* = 37	12–18	100	Pre *33.48 ± 3.54
					Post *29.63 ± 3.16
2.	DG	*n* = 20	12.05 ± 2.28	65	Pre *29.33 ± 1.11
					Post *28.63 ± 0.94
	EG	*n* = 40	12.30 ± 2.61	70	Pre *29.33 ± 1.29
					Post *26.09 ± 1.05
3.	NEF	*n* = 25	7.59 ± 1.26	70.4	1.69 ± 0.62
	ED	*n* = 35	8.41 ± 1.20	63.8	2.00 ± 0.68
4.	OB_NEF_	*n* = 23	7.6 ± 2.6	56.5	1.74 ± 0.28
	OB_ED_	*n* = 20	7.7 ± 2.8	60	1.76 ± 0.31
	CG	*n* = 26	7.3 ± 2.2	58	1.08 ± 0.21

Abbreviations: BMI, body mass index; BMI‐z, body mass index Z‐score; CG, control group; DG, dietary group; ED, endothelial dysfunction; EG, experimental group; NEF, normal endothelial function; OBED, subjects with obesity and endothelial dysfunction; OBNEF, subjects with obesity and normal endothelial function.

^a^ Total number of patients in this study was 67 subjects, but due to various reasons seven subjects were excluded before the intervention, and the next 13 subjects during the intervention phase of experiment.

### 3.2 Non‐alcoholic fatty liver disease

NAFLD is a disease comprising of two main clinical features – steatosis and inflammation of liver. It is a condition resulting from factors other than alcohol consumption.^39^ Studies 5 and 6 examined the correlations of miRNAs levels (miR‐199a‐5p, miR‐122‐5p, and others) with serum levels of clinically approved markers of hepatic steatosis: aspartate‐aminotransferase, alanine‐aminotransferase (both articles), glutamate‐pyruvate‐transaminase (only study 6) (respectively: AST, ALT, and GGT); as well as with BMI and BMI‐z scores of children with obesity (BMI >95th percentile) and NAFLD (NAFLD) in comparison to the ones without obesity and NAFLD (CG) (study 5), or in three paediatric cohorts of children with overweight, recruited from hospitals in Germany (GC), Italy (IC), and Slovenia (SC) (study 6). Additional marker used in study 6 was cytokeratin 18 (CK18) measured in serum or plasma using enzyme‐linked immunosorbent assay (ELISA). In both studies, severity of liver steatosis was graded using ultrasonography (USG) into scales: undetectable/mild/diffuse (study 5) or no‐steatosis/grade I/grade II/grade III (study 6).

### 3.3 Type 2 diabetes mellitus

The goal of study 7 was the comparative analysis of miRNA levels in paediatric groups of children with (OB) or without obesity (CG) and children who were overweight (OW); adult groups of patients with type 2 diabetes/normal glucose tolerance (T2DM/NGT) (total of 352 subjects) and animal model groups of mice with obesity/diabetes (ob/db). First stage was miRNA profiling of nine patients from OB and CG groups each, validated further in full‐size final paediatric groups (study 2) and in groups of adults with T2DM/NGT. MiRNAs which were chosen according to the results of the experiment (miR‐15b, miR‐146b, and miR‐486) were then transfected using lentivirus into human preadipocyte, murine beta‐cell (MIN6), and myoblast (C2C12) cultures in order to examine their influence on respectively: adipocytes cell proliferation, insulin secretion, and glucose intake. Obesity and overweight in children were measured with BMI standard deviations (SDs), according to WHO Child Growth Standards (CGS) – children under 60 months: obesity >3 SDs, overweight >2 SDs; children over 60 months: obesity >2 SDs, overweight >1 SDs. Measure of T2DM was fasting plasma glucose (FPG) >7.0 mmol/L. Information on FPG as well as age, serum triglyceride, and cholesterol levels of subjects were collected from hospitals.

Study 8 was conducted as a response to the results of study 7. Serum levels of three selected miRNAs (miR‐15b, miR‐146b, and miR‐486), as well as of the betatrophin, were measured and compared between the groups of children with (>95th BMI percentile) or without (5th to 85th BMI percentile) obesity, and children suffering from diabetes (classification based on previous results of FPG, blood glucose, oral glucose tolerance test, or glycated haemoglobin [HbA1c]). Venous blood samples intended for measurement of percentage of HbA1c (HbA1c%), betatrophin, cholesterol, glucose, and miRNAs relative expression levels were collected after overnight fasting. Correlations of miRNAs expression, betatrophin, and other parameters were tested by Pearson correlations coefficient (significance: *p* < 0.05). ROC of miRNAs and serum betatrophin were created in order to assess the potential of differentiating between the groups.

These two studies presented, though to a different extent, a profile of three miRNAs (namely miR‐486‐5p, miR‐146a‐5p, and miR‐15b) potentially useful in detection of T2DM development in children. Results of study 8 are less satisfactory than those of study 7, showing lower AUC score values. However, it should be pointed out, that in study 7 the cumulative effect of simultaneous measurement of two or three of aforementioned molecules was not examined.

Another two miRNAs (miR‐29a, miR‐122) were examined in study 9 in terms of differences of their levels in children without obesity, with obesity but without T2DM, and in those with obesity and T2DM. Two hundred and ninety eight patients, aged 9–15 years, were divided into above groups based on their BMI and WC (obesity vs. non‐obesity) and glucose levels (fasting ≥126 mg/dl, random ≥200 mg/dl, OGTT ≥126 mg/dl 2 h post‐ingestion), or HbA1c% >6.5%. The groups were significantly different in terms of diabetes parameters (glucose, insulin, homeostatic model assessment IR [HOMA‐IR]), whereas children with obesity and without T2DM did not differ significantly in WC and BMI percentiles from children with obesity and T2DM.

### 3.4 Metabolic syndrome

Similarly to the obesity, we can observe the increasing metabolic syndrome (MetS) prevalence worldwide. This term represents the cluster of metabolic changes, which stem directly from long‐term positive energy balance. These alterations include obesity, followed by hypertension, and atherogenic dyslipidemia.^40^ Recently the study on 250 adolescents (study 10) investigated the relation between miRNAs and adipokines, specifically adiponectin and leptin. The latter are known to play important role in regulating and maintaining the metabolic homeostasis,^41^ therefore their dysregulation is a strong evidence of developing metabolic disorders. Patients were categorized based on their obesity scores into three groups: without obesity (NW; 50 subjects), suffering from overweight (OW; 100 subjects), and from obesity (OB; 100 subjects).

A potential of miR‐24‐3p molecule in differentiating children with obesity (BMI ≥95th centile according to Working Group on Obesity in China) from children without obesity, as well as children with MetS from ones without MetS, was examined in study 11. The population of study consisted of three groups: children without obesity (*n* = 50); with obesity but without MetS (*n* = 45); with obesity and MetS (*n* = 52). Cut‐off points of metabolic parameters for the MetS were as follows: FBG ≥5.6 mmol/L or type 2 diabetes; SBP ≥130 mmHg and/or DBP ≥85 mmHg; TG ≥1.7 mmol/L; HDL‐c <1.03 mmol/L.

In study 12, a group of 109 children with severe obesity and its complications (hypertonia, T2DM, and MS) were examined in terms of panel of 16 circulating miRNAs (including miR‐122, ‐192, ‐34a), hepatic transaminases, anthropometric, and blood metabolic parameters. Similar to study 10, the associations between the miRNA profile and adiponectin, as well as obesity‐related inflammatory markers levels were investigated. The population of the study varied in age (9–18 years, mean 13.1 ± 2.7 years) with a disproportion for sex (37 male/72 female), therefore the results were adjusted for age and sex, which led to differences between the sexes in significance of correlation of some miRNAs with studied parameters.

### 3.5 Insulin resistance

Three studies (studies 13, 14, and 15) concentrated on the correlation between miRNAs and insulin resistance (IR). In study 13, the children and adolescents aged 10–17 years were divided into two groups: children with obesity (OB; 9 patients) and children with obesity and IR (OB‐IR; 21 patients), based on the HOMA‐IR score (threshold value: >4.00). Their anthropometric parameters and biomarkers values (fasting glucose, leptin, adiponectin, adiponectin/leptin ratio, FFA, HDL‐C, systolic, and diastolic blood pressure) were measured and compared between groups. Forty miRNAs were measured in serum of patients and compared with values of biomarkers and anthropometric parameters.

Study 14 in turn examined the role of miRNA‐27a in the development of IR. For that purpose, group of 45 children with obesity (OB) and group of 45 age‐matched children without obesity (CG) were examined in terms of serum levels of miR‐27a and biochemical parameters: fasting blood glucose, adiponectin, leptin, resistin, subfatin, visfatin, and TNF‐α, IL‐6. The correlations between miRNA‐27a and above biomarkers were calculated. Further research was conducted on animal model, comparing the influence of high‐fat and low‐fat diet, as well as knocking‐out gene for miRNA‐27a on mRNA expressions of IRS‐1 and GLUT4 in skeletal muscle tissue.

Analysis of glucose metabolism parameters and their association with miRNA profile in a group of 58 children with overweight or obesity was conducted in study 15. There was no statistical difference in mean age or insulin levels and HOMA‐IR between the sexes, though the latter parameters were insignificantly higher in girls. Two examined miRNAs: miR‐191‐3p and miR‐375, were also further analysed in terms of interactions with their respective pathway targets.

### 3.6 Atherosclerosis

Relations between BMI, miRNA levels (both circulatory molecules transported in EVs, as well as levels in visceral adipose tissue [VAT]) and cholesterol efflux capacity of THP‐1 derived macrophages were examined in study 16, which was based on population of adolescents (12–19 years) with (all subjects were >99th percentile for age‐adjusted BMI) and without obesity (BMI ≤25 kg/m^2^). Cholesterol efflux capacity reduction leads to creation of foam cells, thus being crucial for the development of atherosclerosis.^42^ Lipoprotein concentrations were measured using nuclear magnetic resonance, followed by calculation of the lipoprotein IR index based on HOMA‐IR data from Multi‐Ethnic Study of Atherosclerosis. Adipocyte‐derived EVs were obtained from serum of female subgroup (chosen as representative for cohort) with further extraction of total ribonucleic acid (RNA). Each participant underwent the bariatric surgery after 2 weeks of protein‐saving diet (1000 kcal/day, 50–60 g protein) and pre‐intervention overnight fasting. VAT collected during this process served as the miRNA source. EVs were applied on THP‐1 macrophages to examine its effect on THP‐1 cholesterol intake. THP‐1 macrophages were also treated with 1 or 3 μg/ml solutions of adipocyte‐derived EVs and separately supplemented RPMI 1640 medium in order to assess the cholesterol efflux. These cells were then used for total RNA isolation, and after reverse transcription and PCR amplification of acquired complementary deoxyribonucleic acid, quantitative analysis of expressed genes was conducted.

## 4 DISCUSSION

Facing the newest progress in techniques of isolation, sequencing, and quantification of nucleic acids (in that case miRNA) from various biological materials, we begin to recognize an important role of miRNAs in modulation of nearly every process in human body. Observing changes of miRNA levels and profiles in various physiological and pathological conditions creates the possibilities to establish new ways of disease diagnosis, treatment, as well as the prediction of their comorbidities and complications. Comparison of miRNA profiles in groups of adults and children with obesity and/or its comorbidities is an important issue for critical assessment of miRNAs as markers reflecting the development of obesity and its comorbidities. These molecules modulate expression of genes involved in metabolic pathways common for both age groups. Dysregulation of given molecule, associated with a comorbidity in one group, should also be observed in the other, with differences in its extent attributable to entering further stages of disease and its associated alterations. Such comparison would be a valid method of evaluating the findings for both age groups (adults and children), nonetheless it exceeds the scope of this review, which concentrated solely on studies conducted in the paediatric population.

Examined miRNAs, their level changes, as well as their significance and AUC score are presented in Table 3. Studies 12 and 15 were not applicable for the purpose of this table, and as such were not included.

**TABLE 3:** Summary of results

Article no.	Comorbidity	Molecule	Compared groups	Status (fold change)	Significance (*p*‐value*)	AUC
1.	ED	miR‐126	Pre‐CG versus Pre‐EG	−1.73	<0.01	‐
			Pre‐EG versus Post‐EG	0.31	<0.01	‐
2.	ED	miR‐320a	Post‐EG versus Post‐DG	1.00	<0.001	‐
			Post‐EG versus Pre‐EG	1.00	<0.001	‐
3.	ED	miR‐125a‐5p	ED versus NEF	−1.33 ± 0.11^b^ (−1.27 ± 0.12)^c^	0.02 (0.01)^c^	‐
		miR‐342‐3p		−1.41 ± 0.08^b^ (−1.22 ± 0.06)^c^	0.03 (0.02)^c^	‐
		miR‐365b‐3p		1.41 ± 0.14^b^ (1.52 ± 0.23)^c^	0.004 (0.001)^c^	‐
4.	ED	miR‐630	ED versus NEF	−0.11	<0.0001	‐
5.	NAFLD	miR‐199a‐5p	NAFLD versus CG	17.18	<0.0001	0.9750^a^
		miR‐122‐5p		12.48	<0.0001	0.9833^a^
6.	NAFLD	miR‐122	NAFLD versus NN (GC)	2.25	<0.05	0.77
			NAFLD versus NN (IC)	0.20	>0.05	0.54
7.	T2DM	miR‐15b	OB versus CG	> 6.00	<0.01	‐
			T2DM versus NGT	6.67	<0.01	0.969^a^
		miR‐146b	OB versus CG	> 6.00	<0.01	‐
			T2DM versus NGT	5.75	<0.01	0.882
		miR‐486	OB versus CG	> 6.00	<0.01	‐
			T2DM versus NGT	9.00	<0.01	0.923^a^
8.	T2DM	miR‐486R	OB versus CG	0.39	0.003	‐
			t2DM versus CG	0.78	<0.001	‐
			t2DM versus OB	0.24	0.001	0.61
		miR‐146bR	OB versus CG	0.24	<0.001	‐
			t2DM versus CG	0.46	<0.001	‐
			t2DM versus OB	0.17	<0.001	0.54
		miR‐15bR	OB versus CG	0.13	<0.001	‐
			t2DM versus CG	0.34	<0.001	‐
			t2DM versus OB	0.18	<0.001	0.62
9.	T2DM	miR‐29a	t2DM versus OB	0.21	<0.001	‐
		miR‐122	t2DM versus OB	0.37	<0.001	‐
10.	MetS	miR‐532‐5p	OB versus CG	−4.59	<0.001	‐
		miR‐423‐5p		−3.32	<0.001	‐
		miR‐520c‐3p		−4.41	<0.001	‐
		miR‐146a		−4.83	<0.001	‐
		miR‐15a		−3.67	<0.001	‐
		miR‐142‐3p		4.06	<0.001	‐
		miR‐140‐5p		2.53	<0.001	‐
		miR‐222		2.36	<0.001	‐
		miR‐143		3.02	<0.001	‐
		miR‐130		2.18	<0.001	‐
11.	MetS	miR‐24‐3p	OB versus CG	0.52	<0.001	0.951
			MetS versus OB	0.43	<0.001	0.890
13.	IR	miR‐30d‐5p	OB‐IR versus OB	0.90	<0.05	‐
		miR‐122‐5p		1.17	<0.05	‐
		miR‐221‐3p		0.22	<0.05	‐
		miR‐215‐5p		0.27	<0.05	‐
14.	IR	miR‐27a	OB versus CG	2.70	<0.05	‐
16.	AS	miR‐3129‐5p	HCE versus LCE	OE^d^	<0.05	‐
		miR‐320d		OE^d^	<0.05	‐

Abbreviations: AUC, area under receiver‐operator curve; CG, control group; ED, endothelial dysfunction; GC, German cohort; HCE, high cholesterol efflux; IC, Italian cohort; IR, insulin resistance; LCE, low cholesterol efflux; MetS, metabolic syndrome; miR, microRNA; NA, not‐applicable; NAFLD, non‐alcoholic fatty liver disease; NEF, normal endothelial function; NGT, adults with normal glucose tolerance; NN, non‐NAFLD; OB, children with obesity; OE, overexpressed; post‐EG, post‐intervention experimental group; pre‐CG, pre‐intervention control group; pre‐EG, pre‐intervention experimental group; T2DM, adults with diabetes; t2DM, children with diabetes; UE, under‐expressed.

^a^ Significant AUC result (>0.9).

^b^ Cardiovascular arrays.

^c^ qRT‐PCR (quantitative reverse transcription polymerase chain reaction) validation.

^d^ Lack of data concerning the exact levels of molecules.

* *p*‐values <0.05 were treated as significant.

### 4.1 Endothelial dysfunction

Condition of endothelium as a whole can be inspected via levels of endothelial products, such as NO and ET‐1, their ratio (NO/ET‐1) or through functional test – RHI measurement, whose score is dependent on endothelial reaction engaging NO.^43^, ^44^ Some miRNAs play an established role in physiology of endothelium, angiogenesis, although they have not been yet applied clinically as the markers of endothelial dysfunction.^45^, ^46^

Physical activity is one of the factors contributing to healthy endothelium. MiRNA‐126 is widely regarded as a molecule linked with the functionality of this tissue and as such was chosen for the subject of study 1. It examined a possible role of this molecule in the mechanism of ED renewal during 6 weeks of interventional lifestyle program (dietary and exercise routine change) in population of adolescents with obesity. Intervention resulted in an increased quality of endothelial condition, associated with statistically significant improvement of RHI and NO/ET‐1 parameters (*p* < 0.01 in *t*‐test comparison of pre‐ vs. post‐intervention results). Simultaneously the miRNA‐126 levels also increased, with positive correlation to BMI decrease and to the growth of RHI and NO/ET‐1 (respectively *r* = 0.50, *p* < 0.05; *r* = 0.69, *p* < 0.05; *r* = 0.68, *p* < 0.05).

Method used for the measurement of RHI – peripheral arterial tonometry – is highly dependent on age and sex of subjects,^47^ therefore the uniformity of groups in this regard is highly required. Unfortunately, in this study the patients' age parameter had a wide range (12–18 years) and the RHI results might be unreliable measure of endothelial condition in that case.

Similar to study 1, study 2 was also an interventional study, which compared the effects of 12‐week dietetic intervention or 12‐week dietetic and exercise intervention on anthropometric measurements, levels of blood indices, ED markers, and metastasis‐associated lung adenocarcinoma transcript 1 (MALAT1)/miR‐320a axis in group of obese adolescents. Expression of miR‐320a increased, and expression of MALAT1 decreased significantly more (2‐fold, *p* < 0.001) in patients who apart from changes in diet changed also their life habits. When comparing levels of these molecules in exercise group before and after 12‐week intervention, the same changes were observed for either of the molecules (*p* < 0.001). Changes in miR‐320a expression correlated negatively with changes in levels of ED markers: VCAM‐1 (*r* = −0.646, *p* < 0.001), ICAM‐1 (*r* = −0.653, *p* < 0.001), and E‐selectin (*r* = −0.856, *p* < 0.001), whereas changes in MALAT1 expression correlated positively with these markers (0.694, *p* < 0.001; 0.683, *p* < 0.001; 0.846, *p* < 0.001). MiR‐320a was also significantly negatively correlated with parameters connected with obesity, including BMI (*r* = −0.027, *p* = 0.029), waist circumference (−0.226, 0.041), hip circumference (−0.267, 0.040), TC (−0.501, 0.008), LDL‐C (−0.499, 0.010), and HOMA‐IR (−0.431, 0.028). This molecule may potentially be considered as the marker of obesity‐related pathological changes in endothelium.

The goal of study 3 was to establish the differences in expression of miRNAs specific for cardiovascular diseases between the groups of children with NEF and the ones with ED using microarray assay. Results were further validated (using qRT‐PCR) for three miRNAs whose expressions were found to be significantly different in groups. The procedure was repeated for groups of children with ED and with NEF, but without obesity, in order to rule out the influence of BMI‐z score. MiRNA profile in these groups was similar to the one in groups with obesity.

Gene mapping revealed putative targets of these three molecules (respectively: 1194; 683; 679 genes, and 31 genes mutual for all three), involved in transforming growth factor β signalling, cytokine‐cytokine receptor interactions, and activin receptor‐like kinase pathways in cardiac myocytes, included in MED13 and MED14 mediator complexes. This study suggested the possible value of aforementioned three miRNAs as endothelial dysfunction indicators, but unfortunately they seem to be unrelated to obesity's involvement in development of this comorbidity.

Study 4 identified significantly underexpressed 4 miRNAs (in order of decreasing expression level: miR‐16‐5p, miR‐451a, miR‐5100, and miR‐630) and one overexpressed (miR‐4665‐3p) in children of OB_ED_ group when compared to subjects of OB_NEF_ group. EVs obtained from patients with ED disrupted integrity of cell membranes in endothelial cell culture and significantly (*p* < 0.001) reduced expression of mRNA coding endothelial NO synthase. Injection of EVs derived from OB_ED_ subjects into murine circulation prolonged Tmax significantly (*p* < 0.001) in juxtaposition to the effects of injection of both CG‐ and OB_NEF_‐derived EVs.

MiRNA‐630 was chosen for further examination due to its highest fold change value. Endothelial monolayer culture was treated with EVs transfected with miR‐630 mimic or inhibitor, while the mixture of two was used for control. Mimic induced restoration of the culture from ED cells, while in NEF cells culture (influenced by the inhibitor) the resistance and *zonula occludens‐*1 distribution were altered. Gene targets specific for miRNA‐630 are engaged in 10 pathways, among them in the ones involved in nuclear factor erythroid 2‐related factor 2 – mediated oxidative stress responses, adenosine monophosphate (AMP) kinase, and tight junction signalling.

Relevance of results of this study can be limited due to relatively small size of examined group. Nonetheless, administration of miR‐630 presented a direct regenerative effect on endothelium (as shown in cell culture and animal model), which suggests its potential properties in terms of endothelial regeneration as part of ED treatment strategy.

### 4.2 Non‐alcoholic fatty liver disease

Diagnosis of NAFLD is based on rating the function and condition of liver with liver‐associated enzymes – ALT, AST, GGT,^48^, ^49^ or through biopsy, which provides information concerning steatosis and fibrosis of this organ. Alternative method is combining measurement of ALT/AST and elastography.^50^ Adequate identification scheme for this disease is highly required, considering its rising occurrence. Recently many studies concentrated on said assessment methods^50^, ^51^ although the use of miRNA markers seems to be yet out of the scope of interest.

Results of study 5 presented a panel of 16 (out of 20 tested) miRNAs that were significantly (*p* < 0.05; >2‐fold change) overexpressed in children with NAFLD when compared to healthy controls. Discriminating potential was examined using ROC, leading to assessment of AUC parameter. Twelve molecules had AUC greater than 0.9, although three molecules: miR‐122‐5p (0.9833), miR‐21‐5p (0.9813), miR‐199a‐5p (0.975), and miR‐23a‐3p (0.9708) had the highest scores. Associations between these markers, BMI, and serum transaminases was also analysed – its results are presented in Table 4.

**TABLE 4:** Relation of miRNAs and obesity/NAFLD indicators in study 5

Molecule	Parameter	*r*‐value	*p*‐value
miR‐223‐3p	BMI	0.39	0.04
miR‐21‐5p	BMI	0.40	0.03
miR‐29a‐3p	BMI	0.61	0.0006
miR‐150‐5p	BMI	0.53	0.003
miR‐103a‐5p	BMI	0.44	0.02
miR‐34a‐5p	AST	0.45	0.047
miR‐122‐5p	AST	0.50	0.02
miR‐122‐5p	ALT	0.60	0.006
miR‐192‐5p	ALT	0.59	0.006

Abbreviations: ALT, alanin acylotransferase; AST, aspartate acylotransferase; BMI, body mass index.

In the cohort of this study the greatest fold change between children with and without NAFLD occurred with miR‐199a‐5p (17,18), followed by third highest value of AUC parameter. There was however no significant correlation between this marker and BMI, ALT, and AST parameters. But bearing in mind its role in preadipocyte proliferation and differentiation, insulin signalling, and correlation with state of fibrosis in human liver, miR‐199a‐5p still poses as a strong candidate for the potential NAFLD development marker.

In the contrast, miR‐122 scored the highest AUC and second highest fold‐change (12.48) between the groups and was also positively related with both AST (*r* = 0.50) and ALT (*r* = 0.60). Previous studies in adults established its protective role in non‐alcoholic steatohepatitis (NASH),^52^ as well as in T2DM^53^ and maturity onset diabetes of the young.^54^

Limitation of this study is the lack of comparative analysis of the children suffering from obesity and NAFLD with otherwise healthy children with obesity – the control group of this study consisted of subjects without obesity (mean BMI: 20.11 ± 2.5). Although the findings might be connected directly to NAFLD, the influence of obesity itself on the results cannot be ruled out.

MiR‐122 was also solely investigated in study 6. in comparison to serum markers of NAFLD – AST, ALT, and CK18. In population of two pre‐pubertal cohorts, German and Italian, miRNA (GC: 0.77; IC: 0.54) and serum markers – CK18 (GC: 0.78; IC: 0.54), ALT (GC: 0.89; IC: 0.70) – had the similar potential of differentiating patients with NAFLD from healthy ones (as measured with AUC).

In both groups the miR‐122 matched the quality of CK18, but scored lower when compared to ALT. MiR‐122 was also found to be positively correlating (*r*) with BMI (GC: 0.25; IC: 0.19), AST (GC: 0.46; IC: 0.36), ALT (GC: 0.45; IC: 0.69), and CK18 (GC: 0.31; IC: 0.23), thus suggesting its efficacy in NAFLD grading. Levels of miR‐122 were significantly different between patients with different grades of NAFLD in GC (*p* < 0.05), although no such significance was found in IC.

Obtaining the data from two different centres bears a risk of results being influenced by differences in methodology, used tools and kits, which must be considered a limitation of this study.

Further validation of these findings is needed to approve these markers for clinical use, which in paediatric population would be desirable for measurement of liver steatosis and fibrosis, due to its non‐invasive character, as opposed to the liver biopsy.^3^

### 4.3 Type 2 diabetes mellitus

Obesity stimulates increase of IR, leading to the fully developed non‐insulin dependent diabetes mellitus.^55^ It does so by chronic low‐grade inflammation of adipose tissue, thus the anti‐inflammatory treatment is able to slow its progress.^56^ Genetic background of this pathological process is not yet clear, and examining this field might enable prevention of the development of T2DM in children suffering from obesity.

In order to establish a profile of miRNAs involved in development of this disease, two comparative analysis of miRNA expression were performed in study 7 – one between children with or without obesity and second between patients with T2DM and adults with NGT. After serum miRNA sequencing, followed by RT‐qPCR validation, expression levels of 8 miRNAs proved to be significantly different in children with obesity. Interestingly, the highest Pearson's *r* values were linked with miRNAs, whose expression increased the most – miR‐486, miR‐146b, and miR‐15b.

In miRNA analysis in T2DM/NGT groups the same 3 miRNAs, which were most increased in children with obesity, turned out to be also the most overexpressed in patients with T2DM, when compared to the ones with NGT (*p* < 0.01). They were also positively correlated to FPG values (miR‐486: *r* = 0.923; miR‐146b: *r* = 0.882; miR‐15b: *r* = 0.969). ROCs and AUC values were calculated for each miRNA, as well as for their combination (AUC scores given in Table 5).

**TABLE 5:** AUC scores of miRNA and miRNA combinations in study 7

Subject	miR‐486‐5p	miR‐146a‐5p	miR‐15b‐5p	miR‐486‐5p/miR‐146a‐5p	miR‐486‐5p/miR‐15b‐5p	miR‐146a‐5p/miR‐15b‐5p
AUC	0.923	0.882	0.969	0.928	0.980	0.971

Abbreviations: AUC, area under receiver‐operator curve; miR, miRNA.

These miRNAs were overexpressed in model mice with obesity and diabetes, in relation to development of these two conditions. They were also found in heart, liver, spleen, pancreas, kidney, white fat, lung, and skeletal muscles of adult C57BL mice. MiR‐486‐5p added to preadipocyte culture increased their proliferation, while miR‐146a‐5p inhibited this process. MiR‐15b had no significant effect. In pancreatic beta‐cell dysfunction assay, insulin synthesis, and secretion were impaired in groups treated with miR‐146a‐5p and miR‐15b‐5p. In contrary, the overexpression of miRNA‐486‐5p resulted in improved glucose uptake in skeletal muscles. This study presented a panel of three miRNA markers, which together might create a promising method of grading risk of T2DM development in children with obesity, although these results require further validation.

Study 8 continued the research on these three miRNAs. After isolation from plasma of subjects, the miRNAs were then processed with RT‐qPCR. The 2^−ΔΔCT^ method was applied in order to calculate the differences of expression levels of miRNAs. The levels of betatrophin were measured using ELISA.

Levels of miRNAs and betatrophin correlated positively with BMI percentile, fasting glucose level and HbA1c%, and negatively with insulin and c‐peptide levels in most of the groups. Even though these results seemed promising, studied miRNAs scored lower in terms of differentiating potential between children with and without diabetes, represented with AUC score (miR‐486: AUC = 0.61; miR‐146b: AUC = 0.54; miR‐15b: AUC = 0.62). It stands in opposition to the results of study 6, where these molecules scored significantly higher, although it is worth mentioning, that the population of the 2018 study was significantly more numerous. Also, the cumulative effect of molecules in differentiating was not established, contrary to the aforementioned study.

MiRNA profiles differed significantly (*p* < 0.001) between the groups of children in study 9, with 0.21 fold change of miR‐29a and 0.37 fold change of miR‐122 between children with obesity and diabetes compared to the ones with obesity but without diabetes. In the whole population of the study the significant (*p* < 0.01) positive correlations between miR‐29a and the metabolic parameters: HbA1c% (*r* = 0.570), glucose (0.654), insulin (0.356), C‐peptide (0.254), and HOMA‐IR (0.651) were observed. Analysis of miR‐122 levels showed similarly significant (*p* < 0.01) correlations between this molecule and the above parameters (respectively: 0.468, 0.412, 0.513, 0.293, 0.631). In patients with obesity the levels of both studied miRNAs were significantly correlated with each other. This study suggests the possible role of these molecules in the pathogenesis of T2DM in children with obesity. Its results might be of high significance, considering the reasonable size of the examined groups, as well as a good design of the groups, allowing differentiation of the influence of obesity and diabetes on the miRNA profiles.

### 4.4 Metabolic syndrome

Statistical analysis of correlation between miRNAs and parameters of obesity and MetS in study 10 identified 5 overexpressed (miR–142‐3p, miR‐140‐5p, miR‐222 miR‐143, and miR‐130) and 5 underexpressed (miR‐532‐5p, miR‐423‐5p, miR‐520c‐3p, miR‐146a, and miR‐15a) molecules, all of which were positively linked with the concentrations of adipokines. The upregulated molecules correlated positively with values of BMI, diabetic, and lipid profile parameters, whereas downregulated ones correlated negatively with them. A difference in levels of obesity‐related parameters (blood pressure, fasting blood glucose, blood insulin, HOMA‐IR, C‐peptide, triglycerides, high‐density lipoprotein cholesterol, and low‐density lipoprotein cholesterol) could be observed between the groups, which reflected the down‐ and upregulation of studied molecules.

In study 11, the analysis of potential of miR‐24‐3p in detection of obesity and MetS, expressed as AUC – respectively 0.951 and 0.890. Levels of this molecule were significantly lower in children without MetS than in those with MetS (*p* < 0.001), whose expression of miR‐24‐3p was highest of all groups. They also correlated with some of the parameters, including FBG (*r* = 0.798, *p* < 0.001), TG (0.773, <0.001), SBP (0.746, <0.001), and DBP (0.623, <0.001), but not with TC, LDL, and HDL (all *p* > 0.05). Though this study is well designed and conducted on group of relatively sound size (n = 147), it failed to prove correlation of miR‐24‐3p levels with parameters of dyslipidemia, which are an important component of MetS. Nonetheless, miR‐24‐3p presented the considerable specificity (71.2%) and sensitivity (95.6%) for detecting MetS, with AUC of 0.890. Further trials are needed to evaluate potential of this molecule.

Results of study 12 showed that miR‐122, previously described as involved in hepatic steatosis, and miR‐192 correlated with TNFα (*r* = 0.25, *p* < 0.05; 0.26, <0.05 respectively), IL‐1Ra (0.24, <0.05; 0.22, <0.05), and procalcitonin (0.45, <0.01; 0.39, <0.01), and negatively with adiponectin (−0.27, <0.01; −0.31, <0.01). Similar correlations were observed for miR‐34a and TNFα (0.22, <0.05), and procalcitonin (0.27, <0.01). It suggests that these three miRNAs may play a role in obesity‐related inflammatory reaction, which is an important feature of MetS pathogenesis. Moreover miR‐33b, ‐193b, and ‐1290 were shown to be associated with lipid parameters and hepatic transaminases, although after adjusting for age and sex, the associations of miR‐193b and miR‐93 with CK‐18, as well as of miR‐33a and TG decreased. Interestingly, miR‐122 was the only miRNA which correlated with HOMA‐IR (0.30, <0.01). An insignificant trend (*p* = 0.10) for this molecule was observed in patients with MetS, whereas miR‐93 expression was significantly lower in these patients than in those without MetS. Similar findings were presented for miR‐192 in patients with metabolically unhealthy obesity (defined as HDL‐C >40 mg/dl, TG ≤150 mg/dl, SBP and DBP ≤90th percentile, and fasting glucose ≤100 mg/dl). This study presented a set of miRNAs, which were shown to be firmly correlated with metabolic pathologies stemming from obesity. Nonetheless, the limitation of the study is inclusion of patients with prediabetes (19.3% patients), T2DM (4.6%), and NAFLD (57.8%). Only a 13.9% of patients suffered from MetS, therefore despite the high association of studied molecules with components of MetS, it is difficult to rule out the influence of the obesity comorbidities other than MetS on the results of the study.

As the MetS is the early state leading to severe changes and comorbidities in organism, an adequate early detection of its development using studied molecules could enable prevention of health worsening at its very beginning.

### 4.5 Insulin resistance

Despite the small size of groups in study 13 (9 patients in OB; 21 in OB‐IR), they were significantly different in terms of HOMA‐IR (*p* < 0.01), fasting insulin (*p* < 0.05), TG (*p* < 0.05), and TG/HDL‐C ratio (*p* < 0.05). Out of 40 examined miRNAs, levels of 12 were significantly different in the patients of OB and OB‐IR groups (miR‐223‐3p, ‐23a‐3p, ‐150‐5p, ‐191‐5p, ‐24‐3p, ‐30d‐5p, ‐122‐5p, ‐30a‐5p, ‐215‐5p, ‐221‐3p, ‐145‐5p, ‐342‐3p). Moreover, four of these molecules (miR‐30d‐5p, miR‐122‐5p, miR‐221‐3p, and miR‐215‐5p) correlated with insulin (*p* < 0.001, *r* = 0.804; 0.002, 0.662; 0.035, 0.610; 0.014, 0.639) and TG/HDL‐C (0.011, 0.544; 0.004, 0.596; 0.002, 0.524; 0.019, 0.656) values. Three of these molecules (miR‐30d‐5p, miR‐122‐5p, and miR‐221‐3p) correlated with values of other parameters of IR, such as HOMA‐IR, homeostasis model assessment‐adiponectin (HOMA‐AD), and adipose insulin resistance (Adipo‐IR) index.^57^, ^58^ Although the results of this study suggest the potential of above molecules in diagnosing IR in adolescents, it must be noted that apart from relatively small groups size, patients were also quite nonuniform in terms of age, which might have influenced the outcomes of the study.

Results of the study 14 showed the correlation of serum miR‐27a levels with values of BMI, fasting blood glucose, serum resistin, IL‐6, and TNF‐α. Moreover, miR‐27a levels differed significantly between OB and CG patients. After 12‐weeks of high‐fat diet, miR‐27a levels were found to be significantly higher in skeletal muscle of animals fed a high‐fat diet than in ones fed a low‐fat diet. Increased expression of miR‐27a correlated with lower IRS‐1 and GLUT‐4 expression in high‐fat diet group, which reflected impairment of insulin signalling. In comparison, IRS‐1 and GLUT‐4 expression was higher in animals fed high‐fat diet, in whom gene for miR‐27a was knocked‐out, although not as high as in low‐fat diet group. To sum up, this study proved correlation of miR‐27a levels with obesity‐ and IR‐related parameters, which was also examined and further proved in animal model. It must be however noted, that examined groups of medium size (45 patients each) lacked the significant difference in fasting blood glucose levels (mean 4.59 ± 0.58 vs. 5.03 ± 0.49 mM), which of all examined parameters is the one that is the most associated with development of IR. Therefore, it is difficult to evaluate if increased miR‐27a levels in OB groups reflect impaired insulin signalling or rather the grade of obesity.

In population of study 15, levels of examined miRNAs (miR‐119‐3p and miR‐375) correlated with HOMA‐IR and insulin level, both when the group was analysed as a whole (*p* < 0.001 for either molecule with both HOMA‐IR and insulin), as well as when the patients were divided according to sex and analysed separately. Both miRNAs were found to interact with Hippo signalling pathway, which is responsible for pancreatic β‐cell proliferation and functionality.^59^ It suggests their plausible influence on development of IR and prospectively T2DM. Nonetheless, limitations of the study, such as small group size, have to be taken into consideration.

### 4.6 Atherosclerosis

Cholesterol efflux is a mechanism of cholesterol transfer from macrophages to high‐density lipoproteins, which exhibits inhibiting role in creation of foam cells, in consequence preventing development of atherosclerotic plaques. ATP‐binding cassette transporters A1/G1 (ABCA1/ABCG1) are involved in this process.^60^, ^61^

Study 16 through the multivariate analysis identified two miRNAs which significantly (*p* < 0.05) correlated with cholesterol efflux capacity (miR‐3129‐5p; miR‐20b), and four which correlated negatively (miR‐9‐5p; miR‐320d; miR‐301a‐5p; and miR‐155‐5p).

Interestingly, each of these molecules targets ABCA1 genes expression. After modulating macrophages with VAT‐derived EVs, expression of cholesterol efflux‐related genes did not differ between the cells modulated by EVs of subjects with and without obesity. Nonetheless, treatment with EVs itself led to differences in expression between experiment and control groups, as well as between the experiment groups treated with different EV concentration (3 and 1 μg/ml).

Although the study identified a panel of miRNAs significantly different levels between the subjects with high and low cholesterol efflux capacity, these results lack the correlation with BMI and obesity in subjects, which is essential in order to treat them as relevant in prediction of risk of developing atherosclerosis in children with obesity.

In total, miRNAs have been identified as potential markers identifying, grading the development and even treating five diseases: ED (miR‐630, influencing the endothelial function; miR‐320a, detection of ED development), NAFLD (miR‐122; grading the liver steatosis), T2DM (miR‐486‐5p, miR‐146a‐5p, miR‐15b; identifying T2DM in children), MetS (miR‐532‐5p, miR‐423‐5p, miR‐520c‐3p, miR‐146a, miR‐15a, miR‐142‐3p, miR‐140‐5p, miR‐222, miR‐143, miR‐130, miR‐24‐3p; evaluation of MetS components), and IR (miR‐30d‐5p, miR‐122‐5p, miR‐221‐3p, miR‐215‐5p, miR‐27a; IR assessment). The potential clinical implications of these molecules seem to be promising and require further examination.

## 5 CONCLUSIONS

Review of literature provided us with a good insight into the possibilities of applying miRNAs in diagnosis and prevention of obesity comorbidities.

- Results of reviewed studies indicated miR‐320a as a potential marker useful in diagnosis of ED, whereas miR‐630 was shown to influence the condition of endothelium, therefore posing as a strong candidate for future therapeutic strategy for ED.
- Two miRNAs: miR‐199a‐5p and miR‐122, presented a good potential in diagnosis of NAFLD and grading its development, however further evaluations are recommended.
- Studies concerning type 2 diabetes mellitus suggested miR‐486, miR‐146b, and miR‐15b as markers of disease progression, although the efficacy of these molecules differed between the studies (AUC: 0.88–0.98 in study 7 vs. 0.54–0.62 in study 8). MiR‐122, associated with NAFLD, and miR‐29a were correlated with levels of glucose metabolism parameters. They may also be involved in development of T2DM.
- MiRNAs examined in studies 10, 11, and 12 showed significant correlation with parameters of metabolic syndrome (BMI, diabetic, and lipid profile parameters), which might be used for early detection and planning adequate prevention of this disease.
- Studies 13, 14, and 15. concentrated on miRNAs significantly associated with the development of IR, suggesting the role of several miRNAs (miR‐27a, −30d‐5p, −122‐5p, −221‐3p, and −215‐5p) in its pathogenesis
- Research on a link between miRNAs and cholesterol efflux capacity, which is an important factor in AS development, returned no satisfying results.

Results of above studies indicate that miRNA might play an important role in diagnosis of obesity comorbidities in paediatric population, especially ED, NAFLD, and T2DM. Possibility of early detection of developing obesity‐related pathologies is crucial for creation of efficient strategy aimed at slowing or even inhibiting their progression.

## CONFLICT OF INTEREST

The authors declare no conflicts of interest.

## AUTHOR CONTRIBUTIONS

Michał Hutny and Jagoda Hofman performed the data collection through database browsing, results filtering, and prepared the initial manuscript. Paweł Matusik and Agnieszka Zachurzok conducted the analysis of the gathered results and revised the manuscript versions. All authors were involved in designing the study, writing the manuscript and had final approval of the submitted version manuscript.
